# To Prevent Hair Turning Gray

**Published:** 1881-12

**Authors:** 


To prevent hair from falling out or turning gray, take a teacupful of dried sage, and boil it in a quart of soft water for twenty minutes. Strain it off and add a piece of borax the size of an English walnut; pulverize the borax. Put the sage tea, when cool, into a quart bottle; add the borax; shake well together, and keep in a cool place. Brush the hair thoroughly and rub the wash well on the head with the hand. Then, after a good hard rubbing, brush the hair well before a fire so it will become dry.

It is a little singular that the oftener barrel-organ tunes are ground the duller they become.

